# Ventilator-associated pneumonia related to extended-spectrum beta-lactamase producing Enterobacterales during severe acute respiratory syndrome coronavirus 2 infection: risk factors and prognosis

**DOI:** 10.1186/s13054-024-04906-2

**Published:** 2024-04-20

**Authors:** Keyvan Razazi, Charles-Edouard Luyt, Guillaume Voiriot, Anahita Rouzé, Marc Garnier, Alexis Ferré, Laurent Camous, Nicholas Heming, Nathanaël Lapidus, Anais Charles-Nelson, Armand Mekontso-Dessap, COVID-ICU Group on behalf of the REVA Network and the COVID-ICU Investigators, Alain Mercat, Pierre Asfar, François Beloncle, Julien Demiselle, Tài Pham, Arthur Pavot, Xavier Monnet, Christian Richard, Alexandre Demoule, Martin Dres, Julien Mayaux, Alexandra Beurton, Cédric Daubin, Richard Descamps, Aurélie Joret, Damien Du Cheyron, Frédéric Pene, Jean-Daniel Chiche, Mathieu Jozwiak, Paul Jaubert, Guillaume Voiriot, Muriel Fartoukh, Marion Teulier, Clarisse Blayau, Erwen L’Her, Cécile Aubron, Laetitia Bodenes, Nicolas Ferriere, Johann Auchabie, Anthony Le Meur, Sylvain Pignal, Thierry Mazzoni, Jean-Pierre Quenot, Pascal Andreu, Jean-Baptiste Roudau, Marie Labruyère, Saad Nseir, Sébastien Preau, Julien Poissy, Daniel Mathieu, Sarah Benhamida, Rémi Paulet, Nicolas Roucaud, Martial Thyrault, Florence Daviet, Sami Hraiech, Gabriel Parzy, Aude Sylvestre, Sébastien Jochmans, Anne-Laure Bouilland, Mehran Monchi, Marc Danguy des Déserts, Quentin Mathais, Gwendoline Rager, Pierre Pasquier, Jean Reignier, Amélie Seguin, Charlotte Garret, Emmanuel Canet, Jean Dellamonica, Clément Saccheri, Romain Lombardi, Yanis Kouchit, Sophie Jacquier, Armelle Mathonnet, Mai-Ahn Nay, Isabelle Runge, Frédéric Martino, Laure Flurin, Amélie Rolle, Michel Carles, Rémi Coudroy, Arnaud W. Thille, Jean-Pierre Frat, Maeva Rodriguez, Pascal Beuret, Audrey Tientcheu, Arthur Vincent, Florian Michelin, Fabienne Tamion, Dorothée Carpentier, Déborah Boyer, Christophe Girault, Valérie Gissot, Stéphan Ehrmann, Charlotte Salmon Gandonniere, Djlali Elaroussi, Agathe Delbove, Yannick Fedun, Julien Huntzinger, Eddy Lebas, Grâce Kisoka, Céline Grégoire, Stella Marchetta, Bernard Lambermont, Laurent Argaud, Thomas Baudry, Pierre-Jean Bertrand, Auguste Dargent, Christophe Guitton, Nicolas Chudeau, Mickaël Landais, Cédric Darreau, Alexis Ferré, Antoine Gros, Guillaume Lacave, Fabrice Bruneel, Mathilde Neuville, Jérôme Devaquet, Guillaume Tachon, Richard Gallot, Riad Chelha, Arnaud Galbois, Anne Jallot, Ludivine Chalumeau Lemoine, Khaldoun Kuteifan, Valentin Pointurier, Louise-Marie Jandeaux, Joy Mootien, Charles Damoisel, Benjamin Sztrymf, Matthieu Schmidt, Alain Combes, Juliette Chommeloux, Charles Edouard Luyt, Frédérique Schortgen, Leon Rusel, Camille Jung, Florent Gobert, Damien Vimpere, Lionel Lamhaut, Bertrand Sauneuf, Liliane Charrrier, Julien Calus, Isabelle Desmeules, Benoît Painvin, Jean-Marc Tadie, Vincent Castelain, Baptiste Michard, Jean-Etienne Herbrecht, Mathieu Baldacini, Nicolas Weiss, Sophie Demeret, Clémence Marois, Benjamin Rohaut, Pierre-Henri Moury, Anne-Charlotte Savida, Emmanuel Couadau, Mathieu Série, Nica Alexandru, Cédric Bruel, Candice Fontaine, Sonia Garrigou, Juliette Courtiade Mahler, Maxime Leclerc, Michel Ramakers, Pierre Garçon, Nicole Massou, Ly Van Vong, Juliane Sen, Nolwenn Lucas, Franck Chemouni, Annabelle Stoclin, Alexandre Avenel, Henri Faure, Angélie Gentilhomme, Sylvie Ricome, Paul Abraham, Céline Monard, Julien Textoris, Thomas Rimmele, Florent Montini, Gabriel Lejour, Thierry Lazard, Isabelle Etienney, Younes Kerroumi, Claire Dupuis, Marine Bereiziat, Elisabeth Coupez, François Thouy, Clément Hofmann, Nicolas Donat, Anne Chrisment, Rose-Marie Blot, Antoine Kimmoun, Audrey Jacquot, Matthieu Mattei, Bruno Levy, Ramin Ravan, Loïc Dopeux, Jean-Mathias Liteaudon, Delphine Roux, Brice Rey, Radu Anghel, Deborah Schenesse, Vincent Gevrey, Jermy Castanera, Philippe Petua, Benjamin Madeux, Otto Hartman, Michael Piagnerelli, Anne Joosten, Cinderella Noel, Patrick Biston, Thibaut Noel, Gurvan L. E. Bouar, Messabi Boukhanza, Elsa Demarest, Marie-France Bajolet, Nathanaël Charrier, Audrey Quenet, Cécile Zylberfajn, Nicolas Dufour, Bruno Mégarbane, Sébastian Voicu, Nicolas Deye, Isabelle Malissin, François Legay, Matthieu Debarre, Nicolas Barbarot, Pierre Fillatre, Bertrand Delord, Thomas Laterrade, Tahar Saghi, Wilfried Pujol, Pierre Julien Cungi, Pierre Esnault, Mickael Cardinale, Vivien Hong Tuan Ha, Grégory Fleury, Marie-Ange Brou, Daniel Zafmahazo, David Tran-Van, Patrick Avargues, Lisa Carenco, Nicolas Robin, Alexandre Ouali, Lucie Houdou, Christophe Le Terrier, Noémie Suh, Steve Primmaz, Jérome Pugin, Emmanuel Weiss, Tobias Gauss, Jean-Denis Moyer, Catherine Paugam Burtz, Béatrice La Combe, Rolland Smonig, Jade Violleau, Pauline Cailliez, Jonathan Chelly, Antoine Marchalot, Cécile Saladin, Christelle Bigot, Pierre-Marie Fayolle, Jules Fatséas, Amr Ibrahim, Dabor Resiere, Rabih Hage, Clémentine Cholet, Marie Cantier, Pierre Trouiler, Philippe Montravers, Brice Lortat-Jacob, Sebastien Tanaka, Alexy Tran Dinh, Jacques Duranteau, Anatole Harrois, Guillaume Dubreuil, Marie Werner, Anne Godier, Sophie Hamada, Diane Zlotnik, Hélène Nougue, Armand Mekontso-Dessap, Guillaume Carteaux, Keyvan Razazi, Nicolas de Prost, Nicolas Mongardon, Nicolas Mongardon, Meriam Lamraoui, Claire Alessandri, Quentin de Roux, Charles de Roquetaillade, Benjamin G. Chousterman, Alexandre Mebazaa, Etienne Gayat, Marc Garnier, Emmanuel Pardo, Lea Satre-Buisson, Christophe Gutton, Elise Yvin, Clémence Marcault, Elie Azoulay, Michael Darmon, Hafid Ait Oufella, Geofroy Hariri, Tomas Urbina, Sandie Mazerand, Nicholas Heming, Francesca Santi, Pierre Moine, Djillali Annane, Adrien Bouglé, Edris Omar, Aymeric Lancelot, Emmanuelle Begot, Gaétan Plantefeve, Damien Contou, Hervé Mentec, Olivier Pajot, Stanislas Faguer, Olivier Cointault, Laurence Lavayssiere, Marie-Béatrice Nogier, Matthieu Jamme, Claire Pichereau, Jan Hayon, Hervé Outin, François Dépret, Maxime Coutrot, Maité Chaussard, Lucie Guillemet, Pierre Gofn, Romain Thouny, Julien Guntz, Laurent Jadot, Romain Persichini, Vanessa Jean-Michel, Hugues Georges, Thomas Caulier, Gaël Pradel, Marie-Hélène Hausermann, Thi My Hue Nguyen-Valat, Michel Boudinaud, Emmanuel Vivier, Sylvène Rosseli, Gaël Bourdin, Christian Pommier, Marc Vinclair, Simon Poignant, Sandrine Mons, Wulfran Bougouin, Franklin Bruna, Quentin Maestraggi, Christian Roth, Laurent Bitker, François Dhelft, Justine Bonnet-Chateau, Mathilde Filippelli, Tristan Morichau-Beauchant, Stéphane Thierry, Charlotte Le Roy, Mélanie Saint Jouan, Bruno Goncalves, Aurélien Mazeraud, Matthieu Daniel, Tarek Sharshar, Cyril Cadoz, Rostane Gaci, Sébastien Gette, Guillaune Louis, Sophie-Caroline Sacleux, Marie-Amélie Ordan, Aurélie Cravoisy, Marie Conrad, Guilhem Courte, Sébastien Gibot, Younès Benzidi, Claudia Casella, Laurent Serpin, Jean-Lou Setti, Marie-Catherine Besse, Anna Bourreau, Jérôme Pillot, Caroline Rivera, Camille Vinclair, Marie-Aline Robaux, Chloé Achino, Marie-Charlotte Delignette, Tessa Mazard, Frédéric Aubrun, Bruno Bouchet, Aurélien Frérou, Laura Muller, Charlotte Quentin, Samuel Degoul, Xavier Stihle, Claude Sumian, Nicoletta Bergero, Bernard Lanaspre, Hervé Quintard, Eve Marie Maiziere, Pierre-Yves Egreteau, Guillaume Leloup, Florin Berteau, Marjolaine Cottrel, Marie Bouteloup, Matthieu Jeannot, Quentin Blanc, Julien Saison, Isabelle Geneau, Romaric Grenot, Abdel Ouchike, Pascal Hazera, Anne-Lyse Masse, Suela Demiri, Corinne Vezinet, Elodie Baron, Deborah Benchetrit, Antoine Monsel, Grégoire Trebbia, Emmanuelle Schaack, Raphaël Lepecq, Mathieu Bobet, Christophe Vinsonneau, Thibault Dekeyser, Quentin Delforge, Imen Rahmani, Bérengère Vivet, Jonathan Paillot, Lucie Hierle, Claire Chaignat, Sarah Valette, Benoït Her, Jennifer Brunet, Mathieu Page, Fabienne Boiste, Anthony Collin, Florent Bavozet, Aude Garin, Mohamed Dlala, Kais Mhamdi, Bassem Beilouny, Alexandra Lavalard, Severine Perez, Benoit Veber, Pierre-Gildas Guitard, Philippe Gouin, Anna Lamacz, Fabienne Plouvier, Bertrand P. Delaborde, Aïssa Kherchache, Amina Chaalal, Jean-Damien Ricard, Marc Amouretti, Santiago Freita-Ramos, Damien Roux, Jean-Michel Constantin, Mona Assef, Marine Lecore, Agathe Selves, Florian Prevost, Christian Lamer, Ruiying Shi, Lyes Knani, Sébastien Pili Floury, Lucie Vettoretti, Michael Levy, Lucile Marsac, Stéphane Dauger, Sophie Guilmin-Crépon, Hadrien Winiszewski, Gael Piton, Thibaud Soumagne, Gilles Capellier, Jean-Baptiste Putegnat, Frédérique Bayle, Maya Perrou, Ghyslaine Thao, Guillaume Géri, Cyril Charron, Xavier Repessé, Antoine Vieillard-Baron, Mathieu Guilbart, Pierre-Alexandre Roger, Sébastien Hinard, Pierre-Yves Macq, Kevin Chaulier, Sylvie Goutte, Patrick Chillet, Anaïs Pitta, Barbara Darjent, Amandine Bruneau, Sigismond Lasocki, Maxime Leger, Soizic Gergaud, Pierre Lemarie, Nicolas Terzi, Carole Schwebel, Anaïs Dartevel, Louis-Marie Galerneau, Jean-Luc Diehl, Caroline Hauw-Berlemont, Nicolas Péron, Emmanuel Guérot, Abolfazl Mohebbi Amoli, Michel Benhamou, Jean-Pierre Deyme, Olivier Andremont, Diane Lena, Julien Cady, Arnaud Causeret, Arnaud De La Chapelle, Christophe Cracco, Stéphane Rouleau, David Schnell, Camille Foucault, Cécile Lory, Thibault Chapelle, Vincent Bruckert, Julie Garcia, Abdlazize Sahraoui, Nathalie Abbosh, Caroline Bornstain, Pierre Pernet, Florent Poirson, Ahmed Pasem, Philippe Karoubi, Virginie Poupinel, Caroline Gauthier, François Bouniol, Philippe Feuchere, Florent Bavozet, Anne Heron, Serge Carreira, Malo Emery, Anne Sophie Le Floch, Luana Giovannangeli, Nicolas Herzog, Christophe Giacardi, Thibaut Baudic, Chloé Thill, Said Lebbah, Jessica Palmyre, Florence Tubach, David Hajage, Nicolas Bonnet, Nathan Ebstein, Stéphane Gaudry, Yves Cohen, Julie Noublanche, Olivier Lesieur, Arnaud Sément, Isabel Roca-Cerezo, Michel Pascal, Nesrine Sma, Gwenhaël Colin, Jean-Claude Lacherade, Gauthier Bionz, Natacha Maquigneau, Pierre Bouzat, Michel Durand, Marie-Christine Hérault, Jean-Francois Payen

**Affiliations:** 1 Hôpitaux Universitaires Henri Mondor, DMU Médecine, Service de Médecine Intensive Réanimation, Assistance Publique-Hôpitaux de Paris (AP-HP), 94010 Créteil, France; 2 IMRB, GRC CARMAS, Faculté de Santé de Créteil, Université Paris Est Créteil (UPEC), 94010 Créteil, France; 3 Service de Médecine Intensive Réanimation, Institut de Cardiologie, Sorbonne-Université, Hôpital Pitié–Salpêtrière, and Sorbonne Université, INSERM, UMRS_1166-ICAN Institute of Cardiometabolism and Nutrition, Assistance Publique-Hôpitaux de Paris (APHP), 47–83, Boulevard de L’Hôpital, 75651 Paris, France; 4 Service de Médecine Intensive Réanimation, Hôpital Tenon, Assistance Publique Hôpitaux de Paris, Sorbonne Université, Paris, France; 5 Inserm U1285, CHU Lille, Service de Médecine Intensive - Réanimation, CNRS, UMR 8576 - UGSF - Unité de Glycobiologie Structurale et Fonctionnelle, Univ. Lille, 59000 Lille, France; 6 GRC29, DMU DREAM, Anesthesiology and Critical Care Medicine Department, Tenon Hospital, Assistance Publique-Hôpitaux de Paris (APHP), Sorbonne University, Paris, France; 7 Intensive Care Unit, Versailles Hospital, Le Chesnay, France; 8 Medical and Surgical Intensive Care Unit, Guadeloupe Teaching Hospital, Antilles-Guyane University, Les Abymes, France; 9 Department of Intensive Care, Hôpital Raymond Poincaré, APHP University Versailles Saint Quentin - University Paris Saclay, Paris, France; 10 Laboratory of Infection and Inflammation - U1173, School of Medicine Simone Veil, INSERM, University Versailles Saint Quentin - University Paris Saclay, Garches, France; 11 FHU SEPSIS (Saclay and Paris Seine Nord Endeavour to PerSonalize Interventions for Sepsis), Garches, France; 12 INSERM, Institut Pierre Louis d’Epidémiologie et de Santé Publique, AP-HP, Saint-Antoine Hospital, Public Health Department, Sorbonne University, 75012 Paris, France; 13 Hôpital Européen Georges Pompidou, Unité d’Épidémiologie et de Recherche Clinique, INSERM, Centre d’Investigation Clinique1418, Module Épidémiologie Clinique, AP-HP (Assistance Publique Hôpitaux de Paris), Paris, France; 14 INSERM, Unité U955, Université Paris Est, 94010 Créteil, France; 15 Service de Medicine Intensive Réanimation, CHU Henri Mondor, 51, Av de Lattre de Tassigny, 94000 Créteil Cedex, France

**Keywords:** COVID-19, ARDS, Nosocomial pneumonia, Ventilator-associated pneumonia, ESBL

## Abstract

**Background:**

Patients infected with the severe acute respiratory syndrome coronavirus 2 (SARS-COV 2) and requiring mechanical ventilation suffer from a high incidence of ventilator associated pneumonia (VAP), mainly related to Enterobacterales. Data regarding extended-spectrum beta-lactamase producing Enterobacterales (ESBL-E) VAP are scarce. We aimed to investigate risk factors and outcomes of ESBL-E related VAP among critically ill coronavirus infectious disease-19 (COVID-19) patients who developed Enterobacterales related VAP.

**Patients and methods:**

We performed an ancillary analysis of a multicenter prospective international cohort study (COVID-ICU) that included 4929 COVID-19 critically ill patients. For the present analysis, only patients with complete data regarding resistance status of the first episode of Enterobacterales related VAP (ESBL-E and/or carbapenem-resistant Enterobacterales, CRE) and outcome were included.

**Results:**

We included 591 patients with Enterobacterales related VAP. The main causative species were *Enterobacter sp* (n = 224)*, E. coli* (n = 111) and *K. pneumoniae* (n = 104). One hundred and fifteen patients (19%), developed a first ESBL-E related VAP, mostly related to *Enterobacter sp* (n = 40), *K. pneumoniae* (n = 36), and *E. coli* (n = 31). Eight patients (1%) developed CRE related VAP. In a multivariable analysis, African origin (North Africa or Sub-Saharan Africa) (OR 1.7 [1.07–2.71], *p* = 0.02), time between intubation and VAP (OR 1.06 [1.02–1.09], *p* = 0.002), PaO_2_/FiO_2_ ratio on the day of VAP (OR 0.997 [0.994–0.999], *p* = 0.04) and trimethoprim-sulfamethoxazole exposure (OR 3.77 [1.15–12.4], *p* = 0.03) were associated with ESBL-E related VAP. Weaning from mechanical ventilation and mortality did not significantly differ between ESBL-E and non ESBL-E VAP.

**Conclusion:**

ESBL-related VAP in COVID-19 critically-ill patients was not infrequent. Several risk factors were identified, among which some are modifiable and deserve further investigation. There was no impact of resistance of the first Enterobacterales related episode of VAP on outcome.

**Supplementary Information:**

The online version contains supplementary material available at 10.1186/s13054-024-04906-2.

## Introduction

Antimicrobial resistance is a leading global health issue. Lower respiratory tract infections alone accounted for more than 400,000 attributable deaths and 1.5 million associated deaths in 2019, worldwide [1]. Among critically ill patients, ventilator associated pneumonia is frequent. The incidence of ventilator-associated pneumonia (VAP) in COVID-19 patients ranges from 30 to 84% [2–4]. Potential explanations for the high incidence of VAP in COVID-19 patients include prolonged invasive mechanical ventilation, the high incidence of acute respiratory distress syndrome (ARDS), lung microbiota alteration, COVID-19-related specific lesions, neuromuscular blocking and administration of treatments which depress the immune system. Additionally, COVID-19 patients were often treated empirically by broad-spectrum antibacterial therapy at ICU admission [5]. The main microbial species involved in VAP were Enterobacterales [3, 4]. Recent studies have reported an increase in the frequency of healthcare-associated infections and antibiotic resistance during the COVID-19 pandemic [6]. VAP-attributable mortality was higher for patients with COVID-19 than non COVID-19, with more than 9% of the overall mortality related to VAP [7].

Data regarding the outcome of VAP related to Enterobacterales according to resistance in a homogeneous group of mechanically ventilated COVID-19 patients are scare. We conducted this study to assess risk factors and prognosis of extended-spectrum beta-lactamase producing Enterobacterales (ESBL-E) related VAP in patients with severe acute respiratory syndrome coronavirus 2 (SARS-CoV-2) pneumonia and who developed a first episode of enterobacterales-related VAP.

## Methods, study design and patients

We performed an ancillary analysis of the COVID-ICU study. COVID-ICU was a multi-center, observational, prospective cohort study conducted in 149 ICUs from 138 centers, across three countries (France, Switzerland, and Belgium) which has been previously been described [8]. Ethical committees in Switzerland (BASEC # 2020-00704), France (French intensive care Society CE-SRLF 20-23) and Belgium (2020-294) approved this study and all patients or relatives were informed that their data were included in the COVID-ICU cohort. In case of refusal, the data were withheld accordingly. This manuscript follows the STROBE statement for reporting cohort studies.

### Study population and data collection

All consecutive patients over 16 years of age admitted to participating ICUs between February 25, 2020, and May 4, 2020, with laboratory-confirmed SARS-CoV-2 infection were included. Patients who had been invasively ventilated for more than 24 h before transfer to one of the participating centers were excluded. Details of the data collected daily over the first 14 days from admission and then on Days 28, 45, 60, and 90 have been described elsewhere [8] and are briefly summarized below. We recorded baseline demographics [age, sex, body mass index (BMI), active smoking, treated hypertension, diabetes], long-term corticosteroids, immunodeficiency, Clinical Frailty Scale, and clinical information and ICU severity scores [Simplified Acute Physiology Score (SAPS) II and Sequential Organ Failure Assessment (SOFA)]. The study investigators recorded time-updated information, respiratory support, arterial blood gas, standard laboratory parameters, use of adjuvant therapies for ARDS, microbiological results of respiratory samples and antibiotics use. Patient vital status (with the exact date of death) was collected by study investigators 90 days after ICU admission, with a phone call to patients or their relatives if they were discharged from hospital before Day 90. Data describing the participating centers, including the number of ICU physicians, nurses, and number of beds, were also collected.

### VAP definition

VAP diagnosis was based on: (1) clinical and radiological suspicion based on criteria established by the European Center of Disease Control [9]; (2) confirmed by at least one positive quantitative microbiological sample defined when culture recovered ≥ 10^6^ CFU/mL for tracheal aspirate, ≥ 10^4^ CFU/mL for broncho-alveolar lavage (BAL), and ≥ 10^3^ CFU/mL for distal protected specimen brush or aspirate [9]; and (3) leading the attending physician to initiate antimicrobial therapy. In addition, pneumonia must have occurred at least 48 h after mechanical ventilation onset. For each positive respiratory sample culture, investigators could identify the micro-organism responsible of the infection within a restricted list: Enterobacterales, *Pseudomonas aeruginosa*, *Acinetobacter baumannii*, *Streptococcus pneumonia*, Group A or B *Streptococcus, Enteroccoccus spp*., methicillin-susceptible *Staphylococcus aureus*, methicillin resistant *Staphylococcus aureus*, *Haemophilus infuenzae*, anaerobes or other. Therefore, “other” denotes all micro-organisms not present in the preceding list and were not specified. Since respiratory cultures may identify multiple microorganisms, investigators could identify several micro-organisms within a single respiratory sample.

For this report, we restricted our analysis to patients who presented VAP related to *Enterobacterales* in whom resistance status (including ESBL-E and CRE) were known. Additional data were requested from participating centers, including involved species and coinfection with other micro-organisms. Only the first episode of VAP was considered. ESBL-E phenotype were determined by disk diffusion. Double-disk diffusion testing of clinical specimens detected production of ESBL by a synergistic effect between clavulanic acid/amoxicillin or clavulanic acid/ticarcillin and cefotaxime, ceftazidime, aztreonam or cefepime.

### Statistical analysis

Categorical variables were expressed as number (percentage) and continuous variables as median and interquartile range [IQR]. When appropriate, chi square and Fisher’s exact tests were used to compare categorical variables. The Mann–Whitney U test and the Wilcoxon test were used to compare continuous variables when applicable. To identify patients’ characteristics associated with ESBL-E VAP, we used multivariable logistic regression. Non-redundant variables selected by bivariate analysis (*p* < 0.10) or considered clinically relevant were entered into a logistic regression model. Multiple imputations were used to replace missing values using chained equations method and five imputations. Overall survival curves were estimated using the Kaplan–Meier method and a Cox model to assess the effect of Enterobacterales on overall survival. Results were expressed as hazard ratio and 95% confidence interval. We used a Fine and Gray model (cumulative incidence function of the Gray model) to properly estimate the effect of Enterobacterales related VAP on weaning, while considering death as a competing event using cmprsk package developed by Gray in R software (http://biowww.dfci.harvard.edu/~gray/cmprsk_2.1-4.tar.gz). Results were expressed as subdistribution hasard ratio associated to its 95% confidence interval. Two-sided *p*-values < 0.05 were considered significant.

## Results

**Fig. 1:**
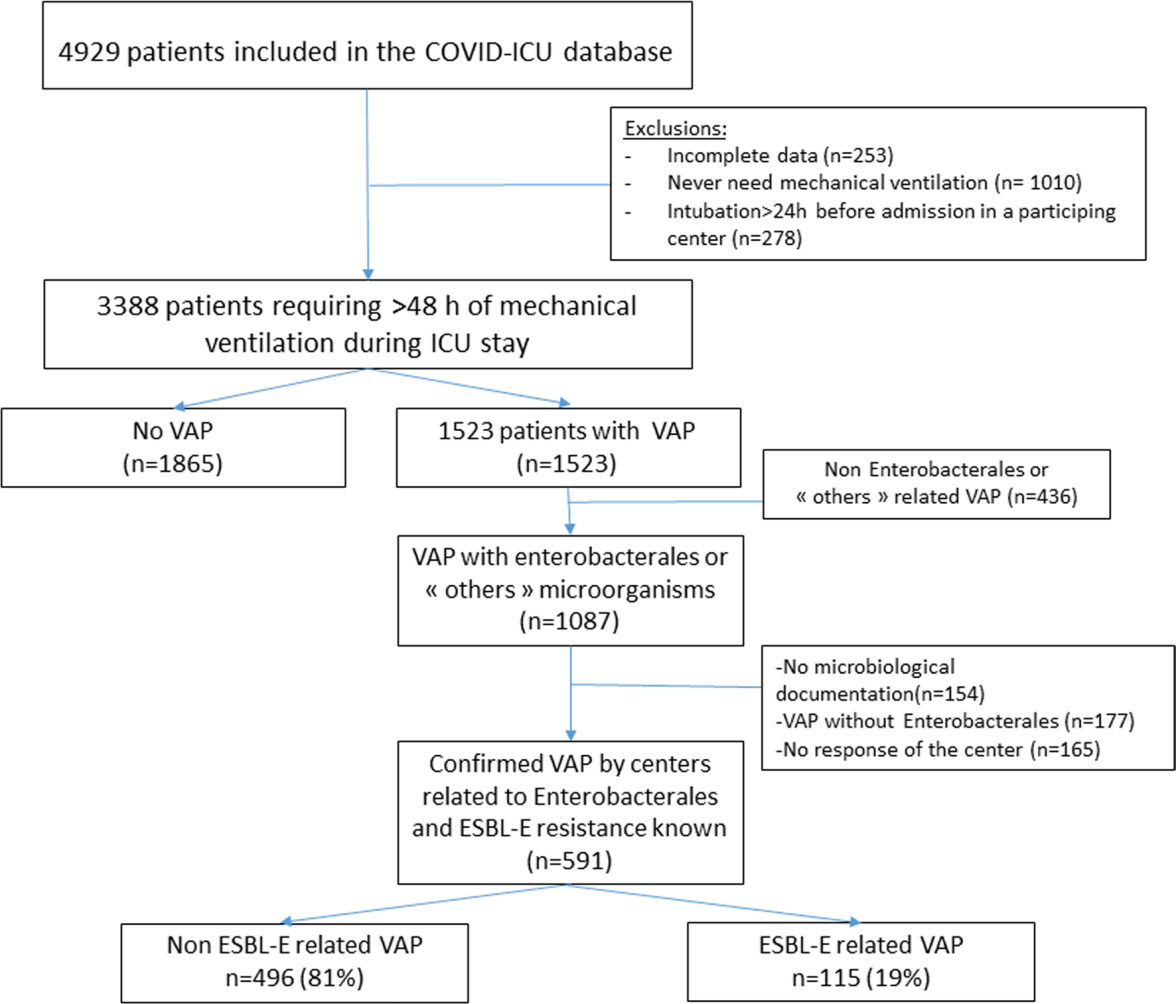
Flow chart of the study

Among the 4929 patients included in the COVID-ICU database, 1087 had VAP with Enterobacterales or “other” microorganism in the database. After additional data requested from participating centers, 494 patients were excluded because respiratory sample found no micro-organism (n = 154), no Enterobacterales but another microorganism (n = 177), or no response of the center (n = 165). 591 had all data available on Enterobacterales related VAP and were included in the current analysis (see flow chart Fig. 1). Characteristics of patients and their clinical and biological data at ICU admission are available in Tables 1 and 2. Median age was 63 [55–69] years. Four hundred and sixty-eight (79%) patients were male. Two hundred and thirty-eight (42%) patients were obese (BMI ≥ 30 kg/m^2^). The most frequent comorbidities were hypertension (298/586, 51%) and diabetes (156/588, 27.5%). There were 46/588 (8%) immunocompromised patients. Median SAPS II and SOFA scores at ICU admission were 37 [28–49] and 5 [3–8], respectively.

**Table 1:** Demographic, clinical, biological and ventilatory support characteristics of patients with VAP according to the occurrence of ESBL-E VAP

	Number with missing data	No ESBL-E VAP (n = 476)	ESBL-E VAP (n = 115)	*P* value
Age, years	0	63 [56–70]	63 [53–68]	0.22
Male gender	3	375 (79%)	93 (81%)	0.71
Body mass index, kg/m^2^	30	29 [25–33]	29 [25–33]	0.65
Ethnic origin	111			0.03
Caucasian		218 (57%)	43 (43%)	
African		111 (29%)	42 (42%)	
Other		52 (14%)	14 (14%)	
Clinical frailty scale	53	2 [2–3]	2 [2–3]	0.66
*Comorbidities*				
No comorbidities	2	81 (17%)	16 (14%)	0.41
Alcohol consumption	15	22 (5%)	5 (5%)	0.92
Tabaco consumption	14	19 (4%)	4 (4%)	0.99
Chronic respiratory disease	5	103 (22%)	28 (25%)	0.49
Cardiovascular co-morbidities	8	306 (65%)	70 (61%)	0.53
Treated hypertension	5	241 (51%)	57 (50%)	0.84
Coronary artery disease	4	52 (11%)	9 (8%)	0.33
Chronic heart failure	8	15 (3%)	4 (4%)	0.77
Known Diabetes	3	127 (27%)	29 (25%)	0.77
Chronic renal failure	3	40 (8%)	16 (14%)	0.07
Cirrhosis	3	3 (1%)	0 (0%)	0.99
Immunodeficiency	3	31 (7%)	15 (13%)	0.02
Hematological malignancies	3	13 (3%)	3 (3%)	0.99
Active solid tumor	3	5 (1%)	0 (0%)	0.59
Solid organ transplant	3	9 (2%)	6 (5%)	0.04
Human Immunodeficiency Virus	3	6 (1%)	2 (2%)	0.66
*Home treatment*				
Immunosuppressive therapy^a^	101	17 (4%)	11 (11%)	0.009
Long-term corticosteroids	102	16 (4%)	8 (8%)	0.09
Treatment with NSAID before ICU admission	91	34 (9%)	7 (7%)	0.63
In another country during 3 weeks before ICU admission	89	15 (4%)	4 (4%)	0.99
Living place: home	3	454 (96%)	114 (99%)	0.15
*At ICU admission*				
SAPS II score	27	37 [29–51]	41 [32–49]	0.25
SOFA score at ICU admission	72	6 [3–9]	7 [4–9]	0.13
*Patient origin*				
Emergency room Direct admission from home/emergency medical ambulance	1	300 (63%)	67 (58%)	
Medical wards	1	130 (27%)	38 (33%)	
Other ICU or Operating theatre	1	45 (9%)	10 (9%)	
Concomitant bacterial pneumonia	11	47 (10%)	9 (8%)	0.49
Invasive mechanical ventilation	3	369 (78%)	89 (77%)	0.89
Biology^c^				
White blood count, × 10^6^/L	44	8600 [6200–11700]	9000 [5300–9800]	0.50
C-reactive protein, mg/L	274	160 [110–248]	169 [113–267]	0.46
Procalcitonine, ng/mL	386	0.46 [0.23–1.37]	0.64 [0.38–2.48]	0.08
During the first 48 h following ICU admission				
Prone position	42	215 (48%)	60 (57%)	0.11
Neuromuscular blockade	42	385 (86%)	96 (91%)	0.19
ECMO	2	24 (5%)	3 (3%)	0.33
Dialysis	2	33 (7%)	14 (12%)	0.06
Antibiotics	1	435 (92%)	109 (95%)	0.25
CT scan	51	113 (26%)	25 (25%)	0.89
Corticosteroids	3	81 (17%)	17 (15%)	0.61
Corticosteroids the first week	0	126 (27%)	31 (27%)	0.98
On the day of VAP				
Time between admission and VAP	0	10 [7–15]	12 [9–21]	0.001
Time between intubation and VAP	0	8 [5–12]	10 [7–18]	0.003
SOFA score	194	9 [7–11]	10 [7–13]	0.15
Non respiratory SOFA	194	7 [7, 8]	7 [4–9]	0.21
Catecholamine	94	153 (37%)	34 (39%)	0.76
PaO_2_/FiO_2_	110	150 [115–220]	138 [103–184]	0.02
PEEP	107	11 [8–13]	12 [10–14]	0.03
Neuromuscular blockade	93	193(47%)	47 (53%)	0.28
Prone position	92	105 (26%)	28 (32%)	0.23
ECMO	85	28 (7%)	8 (9%)	0.43
Dialysis	80	51 (12%)	15 (17%)	0.24
Antibiotics	81	237 (56%)	56 (62%)	0.30

Categorical variables are expressed as n (%) and continuous variables as median [interquartile range]

^a^Except corticosteroids.; NSAID: non-steroidal anti-inflammatory drug; SAPS II Simplified Acute Physiology Score II, SOFA: Sequential Organ Failure Assessment; ECMO extracorporeal membrane oxygenation; VAP ventilator associated pneumonia

**Table 2:** Antibiotics in ICU before VAP related enterobacterales according to ESBL-E

Variables	No ESBL-E VAP (n = 476)	ESBL-E VAP (n = 115)	*P* value
Ab in ICU before VAP	443 (93%)	112 (97%)	0.086
Penicillins	207 (44%)	49 (43%)	0.86
Cephalosporin	340 (71%)	90 (78%)	0.14
Fluoroquinolone	6 (1%)	6 (5%)	0.02
Carbapenem	23 (5%)	9 (8%)	0.20
Aminoglycoside	42 (9%)	13 (11%)	0.41
Co-trimoxazole	7 (1%)	8 (7%)	0.001
Glycopeptides	12 (3%)	8 (7%)	0.02
Linezolid	30 (6%)	6 (5%)	0.66
Macrolides	278 (58%)	67 (58%)	0.98

### ESBL-E VAP

Among the 591 patients with a first episode of VAP related to Enterobacterales, the main species were *Enterobacter sp* (n = 224), *E. coli* (n = 111) and *K. pneumoniae* (n = 104). One hundred and ninety (32%) patients had polymicrobial VAP involving the following species: several Enterobacterales without other species (n = 64, 11%), *Staphyloccocus* *aureus* (n = 31, 5%), *Streptococcus sp.* (n = 20, 3%), *Haemophilus influenzae* (n = 12, 2%), *P. aeruginosa* (n = 42, 7%), or other non-fermenting gram negative bacteria (n = 17; 3%). One hundred and fifteen patients (19%), developed a first episode of VAP with ESBL-E, mostly caused by *Enterobacter sp* (n = 40), *K. pneumoniae* (n = 36), and *E. coli* (n = 31). Eight patients (1%) developed CRE related VAP. All but one CRE VAP were also ESBL-E and were therefore analyzed within the ESBL-E VAP group. There was no significant association between ICU characteristics and ESBL-E VAP, except for a higher number of ICU beds in the ESBL-E group (Additional file 1: Table E1). Risk factors for developing ESBL-E VAP were tested by univariate analysis (Tables 1, 2). African origin (North Africa or Sub-Saharan Africa), time from intubation, immunodeficiency, lower oxygenation, and higher positive end expiratory pressure level, were associated with ESBL-E VAP. Concerning antibiotic exposure, risk factors for ESBL-E VAP included exposure to fluoroquinolone, trimethoprim-sulfamethoxazole or glycopeptid. By multivariable analysis (Table 3), African origin (OR 1.7 [1.07–2.71], *p* = 0.02), time between intubation and VAP (OR 1.06 [1.02–1.09], *p* = 0.002), PaO_2_/FiO_2_ ratio on the day of VAP (OR 0.997 [0.994–0.999], *p* = 0.04) and Trimethoprim-sulfamethoxazole exposure (OR 3.77 [1.15–12.4], *p* = 0.03), were associated with ESBL-E VAP. Risk factors did not differ after excluding the eight patients with CRE VAP, excepted for PaO_2_/FiO_2_ ratio on the day of VAP which felt short of statistical significance [OR = 0.99 (0.99–1.00), *p* = 0.07) (Additional file 1: Table E2). During the 24 h following VAP onset, patients with ESBL-E VAP received more frequently carbapenem [26/115 (23%) vs 49/476 (10%), *p* < 0,001] and less frequently penicillins [27/115(23%) vs 164/476 (34%), p = 0.02], as compared to their counterparts (Additional file 1: Table E3).

**Table 3:** Multivariable analysis of risk factors of ESBL-E related VAP

Variables	OR	95% CI	*p*-value
SAPS II at ICU admission	1.00	0.98–1.01	0.90
African origin	**1.70**	**1.07–2.71**	**0.02**
Chronic renal failure	1.31	0.61–2.78	0.48
Immunodeficiency	1.38	0.62–3.09	0.42
Time between intubation and VAP	**1.06**	**1.02–1.09**	**0.002**
Non respiratory SOFA*	1.05	0.92–1.20	0.48
PaO_2_/FiO_2_*	**0.997**	**0.994–0.999**	**0.04**
ECMO*	0.69	0.29–1.64	0.41
Antibiotics in ICU before VAP	2.23	0.63–7.90	0.21
Fluoroquinolone before VAP	2.59	0.71–9.43	0.14
Co-trimoxazole	**3.77**	**1.15–12.4**	**0.03**
Glycopeptides	1.65	0.58–4.67	0.35

Bold value indicates *p* < 0.05

African origin denotes North Africa or Sub-Saharan Africa origin; SAPS II Simplified Acute Physiology Score II, SOFA: Sequential Organ Failure Assessment; ECMO extracorporeal membrane oxygenation; VAP ventilator associated pneumonia, ICU intensive care unit *on the day of VAP

### Outcome

**Fig. 2:**
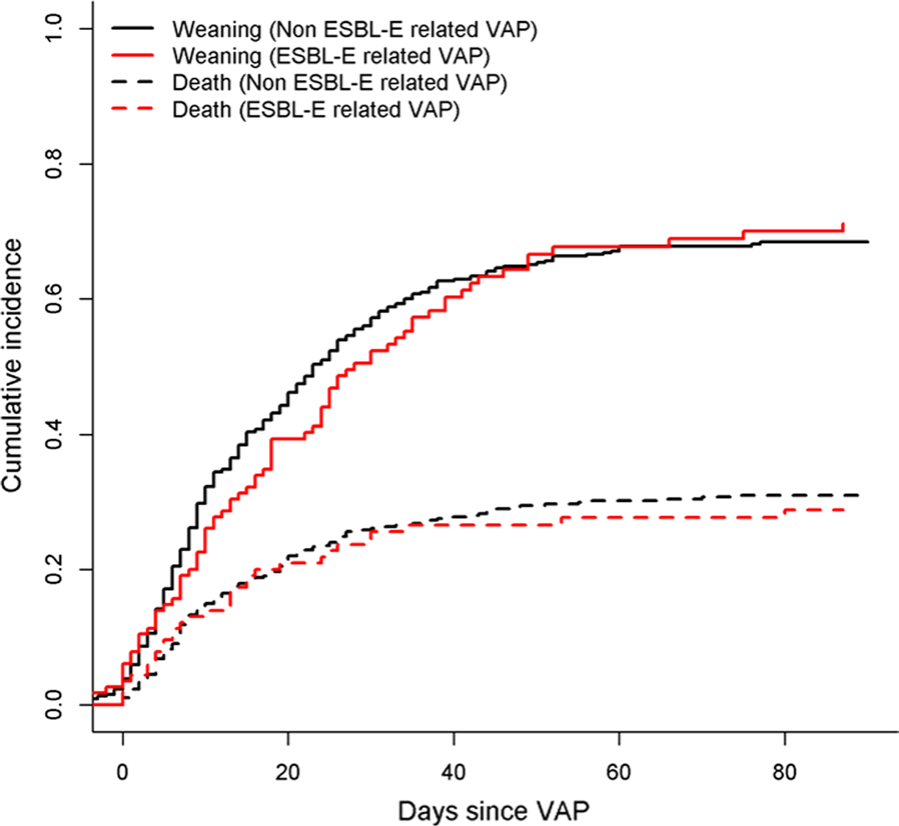
Cumulative probability of weaning in ESBL-E related VAP (red) and non ESBL-E related VAP (black) patients. For analysis purpose, time from intubation to weaning (continuous line) to death (dotted line) were handled as competing risks

**Fig. 3:**
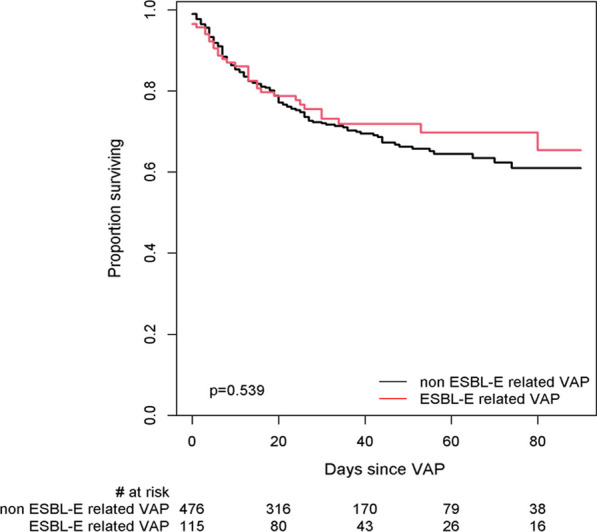
Ninety-day survival in patients with ESBL-E related VAP and non ESBL-E related VAP

The number of VAP episodes after the first episode was lower in patients with VAP due to ESBL-E or CRE as compared to patients with non-ESBL-E VAP (1 [0–1] vs 1 [0–2], p = 0.04). ESBL-E VAP was not associated with a worse outcome as compared to non-ESBL-E VAP. Weaning from mechanical ventilation (Fig. 2), as well as mortality at ICU discharge, in hospital, at 28 days and at 90 days (Additional file 1: Table E4, Fig. 3) did not differ between ESBL-E and non ESBL-E VAP. Mortality was not influenced by species, chromosomally-encoded AmpC-producing Enterobacterales, or polymicrobial VAP (Additional file 1: Table E5). Results on mortality were similar after excluding the eight patients with CRE VAP (HR 0.94 [0.62–1.41], *p* = 0.75), after excluding polymicrobial VAP with *Staphylococcus aureus* or *Pseudomonas aeruginosa* (HR 0.91 [0.61–1.36], *p* = 0.64), after excluding polymicrobial VAP with other species than Enterobacterales (HR 0.94 [0.62–1.41], *p* = 0.75), in 220 patients sampled with BAL or protected distal sample (HR 1.07 [0.62–1.86], *p* = 0.81), or after adjusting for the use of corticosteroids in the first week (HR 0.89 [0.61–1.3], *p* = 0.54).

## Discussion

The main findings of this prospective multicenter study of patients with a first episode of Enterobacterales related VAP, during the first COVID wave are: (i) 19% of VAPs were related to ESBL-E; (ii) African origin, duration of mechanical ventilation, PaO2/FiO2 ratio the day of VAP and trimethoprim-sulfamethoxazole exposure were associated with ESBL-E VAP; (iii) ESBL-E VAP was not associated with a worse outcomes, including mortality, as compared to other Enterobacterales VAP.

Resistance patterns of Enterobacterales related VAP were heterogeneous across studies in COVID-19 patients. Our finding of 19% ESBL-E among Enterobacterales VAP is in accordance with findings from Vacheron et al., describing a frequency of ESBL-E VAP in COVID-19 or non-COVID-19 patients of about 13% in France [10]. Prevalence of ESBL-E ICU acquired infection were reported as heterogeneous in France [11]. Most patients in our study were recruited in regions with higher ESBL-E prevalence (58% of the patients were recruited in the Paris or Greater Paris area).

ESBL-E infection is usually associated with previous colonization and invasive procedures [12]. Thus, it was not surprising that time between intubation and VAP was one of the main risk factor of ESBL-E VAP, as previously shown [13]. A previous study also showed that the prevalence of ESBL increased during the second or third episode of VAP [2]. In our study, microbiological details were only available for the first episode of VAP [2]. In our cohort, most patients received antibiotics before VAP occurrence and the use of trimethoprim/sulfamethoxazole before VAP onset was independently associated with ESBL-E VAP. Exposure to trimethoprim/sulfamethoxazole was associated with ESBL-E organisms in previous reports [13–16]. The third risk factor identified by multivariable analysis was an African origin. This is in accordance with a previous French study reporting that a country of birth outside of Europe was a risk factor for ESBL-E infection [17]. In the USA, an African American origin was reported as a risk factor for ESBL-E infection [14, 18]. The role of a patient’s origin may be driven by diet, food habits and/or travel abroad over the previous years [19]. We could only assess travels over a 3 week period preceding ICU admission. A lower PaO_2_/FiO_2_ ratio on the day of VAP was also a risk factor for ESBL-E VAP identified in this cohort. These results are in accordance with previous studies showing that ARDS was a risk factor for multidrug resistant bacteria VAP [20].

VAP was associated with significantly increased 28-day mortality in SARS-CoV-2 patients [21]. VAP-attributable mortality was higher for patients with COVID-19, with more than 9% of the overall mortality related to VAP [7]. Current evidence of the clinical burden of infections caused by ESBL-producing bacteria is highly heterogeneous and based mainly on unadjusted estimates derived from retrospective studies [22]. Lambert et al. showed that the risk of death associated with antimicrobial resistance (i.e., additional risk of death to that of the infection) was 1.2 (1.1–1.4) for pneumonia but this effect was mainly driven by *S. aureus* and *P. aeruginosa* [23]*.* We herein did not find different outcomes between ESBL-E and non ESBL-E VAP. Additionally, a sensitivity analysis in patients infected only with Enterobacterales excluded the hypothesis that co-infecting microorganisms could have influenced the results. Several studies with fewer ESBL-E episodes also showed no difference in outcome between ESBL-E and non ESBL-E VAP [24, 25]. Our study with a homogeneous population of COVID-19 patients strengthens these findings. Multi-drug resistant related VAP was associated with increased mortality when empiric antibiotherapy was inadequate [26, 27]. Information on early adequate regimen was unavailable but could be similar between ESBL-E and non ESBL VAP, explaining the absence of difference in outcome in this study. Given the poor accuracy of chest radiograph to detect new infiltrates, the diagnosis of VAP in patients with ARDS is challenging [28]. In a restrictive antibiotic policy, physician may have started antibiotic therapy after culture results. We cannot formally exclude that patients developed ventilator associated tracheobronchitis and not VAP, but the number of patients requiring catecholamine (more than 1/3) was higher than reported in recent VAP cohorts [29]. In addition, mortality was similar in ESBL-E and non ESBL-E VAP among patients with distal quantitative samples. Lastly, mortality in COVID-19 critically was mainly altered by age, comorbidities, corticosteroids and organ failure [30, 31].

Strengths of our study include the large number of patients assessed and data recorded prospectively, and the absence of case-mix with only COVID-19 patients. We acknowledge several limitations to our study. First, all patients were included during the first epidemic wave of SARS-CoV-2 affecting Europe in the spring of 2020, a unique period when ICUs were overwhelmed. It cannot be excluded that antibiotic stewardship in COVID-19 patients with less antibiotic administration at ICU admission may change ESBL-E prevalence. Moreover, the acquisition of immunity following subsequent epidemic waves or vaccination, and/or the emergence of new SARS-CoV-2 variants, may change some of our results. Second, although this study was conducted in 149 ICUs from 138 centers, across three countries, our results were obtained from a west European population, a region of the world with relatively low prevalence of ESBL-E colonization or infection.

## Conclusions

In this prospective multicenter study of patients with a first episode of VAP related to Enterobacterales, almost a fifth were ESBL-E. African origin, duration of mechanical ventilation, a lower PaO_2_/FiO_2_ ratio and trimethoprim-sulfamethoxazole exposure were associated with ESBL-E VAP. ESBL-E VAP was not associated with a worse outcome as compared to other Enterobacterales-related VAP.

### Supplementary Information

**Additional file 1. Table E1.** Centers informations; **Table E2.** Multivariable analysis of risk factors of ESBL-E related VAP after exclusion of the 8 patients with CRE related VAP; **Table E3.** Antibiotics administered in the 24 hours following VAP according to ESBL-E; **Table E4.** Outcome according to the occurrence of ESBL-E VAP; **Table E5.** Risk factors for death in patients with VAP related to enterobacterales according to species.

## Data Availability

The data sets generated during the current study are available from the corresponding author on reasonable request.
