# Supplementary material for: Ventilator-associated pneumonia related to extended-spectrum beta-lactamase producing Enterobacterales during severe acute respiratory syndrome coronavirus 2 infection: risk factors and prognosis
Source: Crit Care. 2024 Apr 20;28:131. doi: 10.1186/s13054-024-04906-2 (PMC11031867; doi:10.1186/s13054-024-04906-2)
Supplement: Supplementary file 1 — Additional file 1. Table E1. Centers informations; Table E2. Multivariable analysis of risk factors of ESBL-E related VAP after exclusion of the 8 patients with CRE related VAP; Table E3. Antibiotics administered in the 24 hours following VAP according to ESBL-E; Table E4. Outcome according to the occurrence of ESBL-E VAP; Table E5. Risk factors for death in patients with VAP related to enterobacterales according to species. [file 13054_2024_4906_MOESM1_ESM.docx]

**Online supplement**

**Ventilator-associated pneumonia related to extended-spectrum beta-lactamase producing Enterobacterales during severe acute respiratory syndrome coronavirus 2 infection: risk factors and prognosis**

Keyvan Razazi^1,2^, MD, PhD, Charles‑Edouard Luyt^3†^, MD, PhD, Guillaume Voiriot ^4†^, MD, PhD; Anahita Rouzé^5†^ MD; Marc Garnier^6^, MD; Alexis Ferré^7^, MD; Laurent Camous^8^, MD; Nicholas Heming^9^, MD; Nathanaël Lapidus^10^, MD, Anais Charles-Nelson^11*^, MD, Armand Mekontso-Dessap^1,2,12*^ MD, PhD COVID-ICU Group on behalf of the REVA Network and the COVID-ICU Investigators.

**Table E1 Centers informations**

|  |  | **ESBL-E VAP** | |  |
| --- | --- | --- | --- | --- |
| **Variables** | **Number with missing data** | **No**  **(n=476)** | **Yes**  **(n=115)** | **P value** |
| **Localisation** | 23 |  |  | 0.16 |
| Great Paris region | 23 | 263 (58%) | 66 (58%) |  |
| North and East of France | 23 | 40 (9%) | 16 (14%) |  |
| Other | 23 | 183 (32%) | 152 (33%) |  |
| **Type of hospital** | 32 |  |  |  |
| Universitary | 32 | 243(54%) | 51 (48%) |  |
| Public general | 32 | 161 (35%) | 38 (36%) |  |
| Private | 32 | 48 (11%) | 18 (17%) |  |
| Number of bed in hospital | 97 | 716 [535-1038] | 730 [505-1200] | 0.95 |
| **Type of ICU** | 31 |  |  | 0.41 |
| Medical | 31 | 180 (40%) | 35 (33%) |  |
| Surgical | 31 | 38 (8%) | 10 (9%) |  |
| Mix | 31 | 235 (52%) | 62 (58%) |  |
| Number of bed in the ICU | 73 | 22 [16-32] | 25 [18-38] | 0.04 |
| **Health care workers** |  |  |  |  |
| Physician | 85 | 8 [6-12] | 9 [6-12] | 0.16 |
| Resident | 85 | 7 [6-10] | 8 [5-10] | 0.58 |
| Nurses | 94 | 45 [34-69] | 48 [34-83] | 0.27 |

**Table E2. Multivariable analysis of risk factors of ESBL-E related VAP after exclusion of the 8 patients with CRE related VAP**

| **Variables** | **OR** | **95% CI** | **p-value** |
| --- | --- | --- | --- |
| **SAPS II at ICU admission** | 1.00 | 0.98-1.02 | 0.86 |
| **African origin** | **1.73** | **1.08-2.80** | **0.02** |
| **Chronic renal failure** | 1.37 | 0.64-2.93 | 0.41 |
| **Immunodeficiency** | 1.43 | 0.64-3.21 | 0.38 |
| **Time between intubation and VAP** | **1.04** | **1.00-1.08** | **0.03** |
| **Non respiratory SOFA*** | 1.06 | 0.93-1.21 | 0.42 |
| **PaO_2_/FiO_2_*** | **0.997** | **0.99-1.00** | **0.07** |
| **ECMO*** | 0.76 | 0.32-1.81 | 0.54 |
| **Antibiotics in ICU before VAP** | 2.12 | 0.60-7.50 | 0.24 |
| **Fluoroquinolone before VAP** | 2.68 | 0.73-9.75 | 0.14 |
| **Co-trimoxazole** | **3.98** | **1.21-13.04** | **0.02** |
| **Glycopeptides** | 1.75 | 0.60-5.10 | 0.31 |

African origin denotes North Africa or Sub-Saharan Africa origin; SAPS II Simplified Acute Physiology Score II, SOFA: Sequential Organ Failure Assessment; ECMO extracorporeal membrane oxygenation; VAP ventilator associated pneumonia, ICU intensive care unit *on the day of VAP

**Table E3. Antibiotics administered in the 24 hours following VAP according to ESBL-E**

| **Variables** | **No ESBL-E VAP**  **(n=476)** | **ESBL-E VAP**  **(n=115)** | **P value** |
| --- | --- | --- | --- |
| ***Ab in 24 hours following VAP*** |  |  |  |
| Penicillins | 164 (34%) | 27 (23%) | 0.02 |
| Cephalosporin | 134 (28%) | 24 (21%) | 0.11 |
| Fluoroquinolone | 8 (2%) | 1 (1%) | 0.99 |
| Carbapenem | 49 (10%) | 26 (23%) | <0.001 |
| Aminoglycoside | 54 (11%) | 14 (12%) | 0.80 |
| Co-trimoxazole | 11 (2%) | 3 (2%) | 0.74 |
| Glycopeptides | 11 (2%) | 4 (3%) | 0.51 |
| Linezolid | 24 (5%) | 2 (2%) | 0.20 |

**Table E4. Outcome according to the occurrence of ESBL-E VAP**

|  |  | **ESBL-E VAP** | |  |
| --- | --- | --- | --- | --- |
| **Variables** | **Number with missing data** | **No**  **(n=476)** | **Yes**  **(n=115)** | **P value** |
| ICU mortality | 0 | 142 (30%) | 30 (26%) | 0.25 |
| Day-28 mortality | 0 | 122 (26%) | 27 (23%) | 0.25 |
| Day-60 mortality | 0 | 140 (29%) | 31 (27%) | 0.27 |
| Day-90 mortality | 0 | 143 (30%) | 32 (28%) | 0.32 |

**Table E5 . Risk factors for death in patients with VAP related to enterobacterales according to species**

| **Variables** | **Death** |  | **HR** | **95% CI** | **p-value** |
| --- | --- | --- | --- | --- | --- |
|  | **No** | **Yes** |  |  |  |
|  |  |  |  |  |  |
| ***E. coli*** | 85 (76.6%) | 26 (23.4%) | 0.77 | [0.51-1.17] | 0.228 |
| ***K. pneumoniae*** | 73 (70.2%) | 31 (29.8%) | 1.01 | [0.69-1.49] | 0.961 |
| ***Enterobacter sp*** | 150 (67.0%) | 74 (33.0%) | 1.28 | [0.95-1.74] | 0.105 |
| **Other*** | 151 (71.6%) | 60 (28.4%) | 0.94 | [0.69-1.28] | 0.683 |
|  |  |  |  |  |  |
| **Chromosomally-encoded AmpC-producing Enterobacterales ^#^** | 235 (68.7%) | 107 (31.3%) | 1.21 | [0.89-1.64] | 0.231 |
|  |  |  |  |  |  |
|  |  |  |  |  |  |
| **Polymicrobial VAP** | 143 (75.3%) | 47 (24.7%) | 0.76 | [0.55-1.07] | 0.113 |
| With *Staphylococcus aureus* | 23 (71.9%) | 9 (28.1%) | 1.02 | [0.52-1.99] | 0.958 |
| With *Pseudomonas aeruginosa* | 29 (69.0%) | 13 (31.0%) | 0.99 | [0.56-1.75] | 0.979 |
|  |  |  |  |  |  |

*Others denotes *Citrobacter sp* (n=45), *Hafnia alvei* (n=40), *Klebsiella oxytoca* (n=28), *Morganella morganii* (n=28), *Proteus sp* (n=33), *Serratia marcescens (n=46);* the possibility of several enterobacterales explains a total exceeding 151.

**^#^** Chromosomally-encoded AmpC-producing Enterobacterales denotes *Enterobacter cloacae, Klebsiella aerogenes, Serratia marcescens, Citrobacter freundii, Providencia spp., Hafnia alvei, and Morganella morganii*
